# A complex surgery of spinal tuberculosis with a psoas abscess accompanied by fibula autografting: an alternative treatment of Pott’s disease

**DOI:** 10.1093/jscr/rjac635

**Published:** 2023-01-10

**Authors:** Marah Mansour, Nour Tanta, Ghina Ismail, Tamim Alsuliman, Issam Salman

**Affiliations:** Faculty of Medicine, Tartous University, Tartous 0000, Syrian Arab Republic; Faculty of Medicine, Damascus University, Damascus, Syrian Arab Republic; Faculty of Medicine, Tartous University, Tartous 0000, Syrian Arab Republic; Hôpital Saint Antoine, Rue de Faubourg Saint Antoine, Paris 75012, France; Department of Neurosurgery, AlHikma Hospital, Tartous 0000, Syrian Arab Republic

**Keywords:** Pott’s disease, spinal TB, psoas abscess, tuberculous spondylitis, *Mycobacterium tuberculosis*, posterior-only approach, fibula autografting, tuberculosis

## Abstract

The most common manifestation of skeletal tuberculosis is tuberculosis spondylitis. Symptoms may progress insidiously from back pain to cause many severe complications. Early diagnosis and management of spinal tuberculosis have special importance in prevention. We report a case of a 24-year-old female who was diagnosed with tuberculous spondylitis, complicated with psoas abscess and grade 1/5 of lower limb weakness. The patient was treated with anti-tuberculous drugs and underwent surgical debridement, interbody fusion and internal fixation accompanied by fibular autografting using a posterior-only approach and supplemental posterior spinal stabilisation on an infected background. Within 14 years of follow-up, full bone graft spinal fusion has been achieved with no major complications. According to its clinical efficacy and feasibility, this procedure is suggested to be an alternative treatment for Pott’s disease.

## INTRODUCTION

Pott’s disease is one of the earliest known human infectious diseases triggered by *Mycobacterium tuberculosis* (TB) [1]. The musculoskeletal apparatus is the third most common site of extra-pulmonary tuberculosis (EPTB) following pleural and lymphatic disease, accounting for 10–35% of all EPTB cases. TB spondylitis is the most common manifestation of skeletal TB, representing approximately 50% of all cases of skeletal TB [2]. Immunosuppression and human immunodeficiency virus infection may be significant risk factors for TB [1]. It usually occurs in the thoracic and lumbar regions of the spine. Clinically, back pain is the most common initial symptom, which may progress slowly and insidiously [2]. It may develop three major clinical features: cold abscesses, neurologic deficits and long-term kyphotic deformity [2, 3]. Neural involvement may cause irreversible damage if not promptly and adequately treated [1]. Thus, early diagnosis and management have special importance in preventing complications such as spinal cord compression and deformities. This article describes a TB spondylitis case, complicated with an abscess.

## CASE PRESENTATION

**Figure 1:**
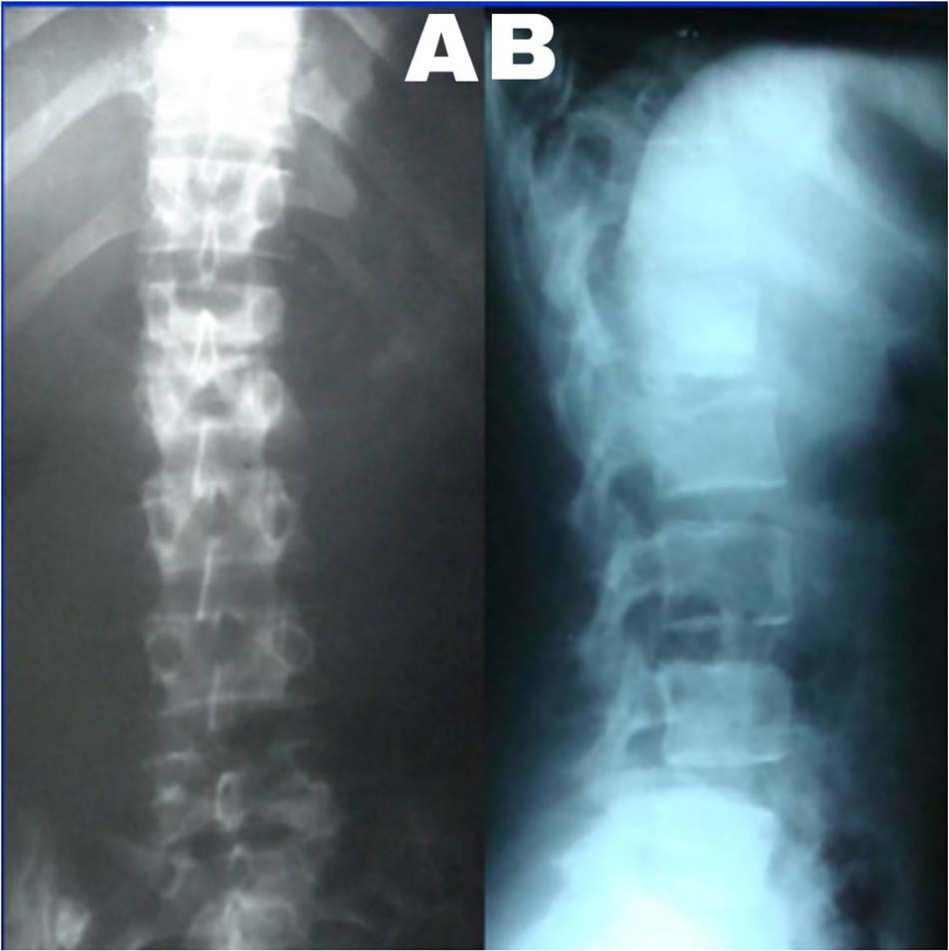
(**A**) Lumbar spine X-ray, anteroposterior view showed the collapse of the L1–2 intervertebral space. (**B**) Lumbar spine X-ray and lateral view showed L2 superior endplate erosion.

**Figure 2:**
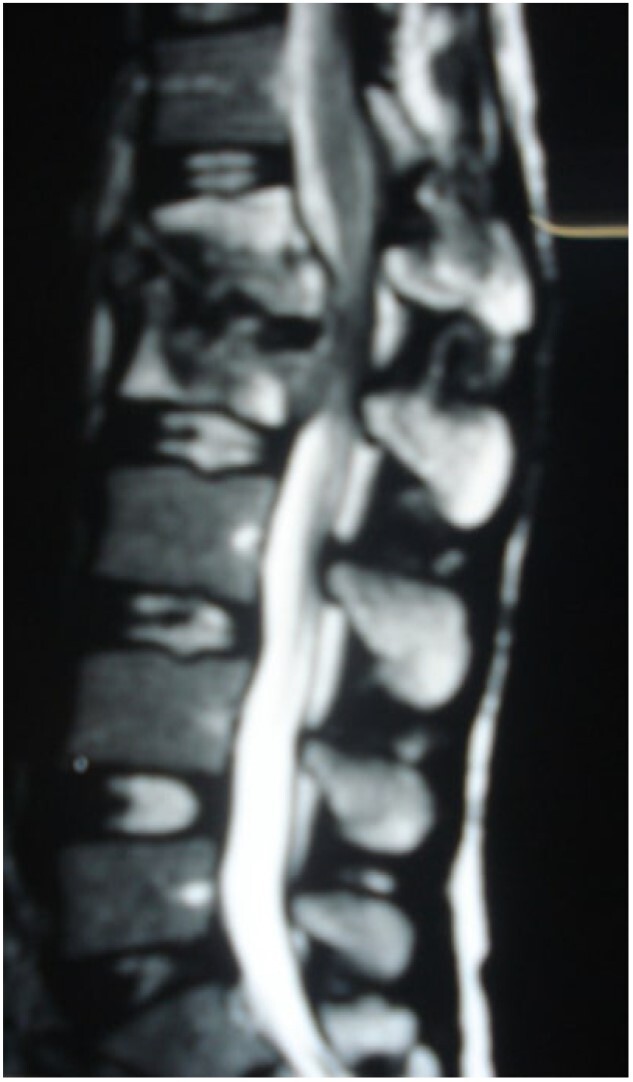
Sagittal T2-weighted MRI shows severe disco vertebral destruction, the formation of epidural abscess and compression of the spinal cord at the L1–2 level.

**Figure 3:**
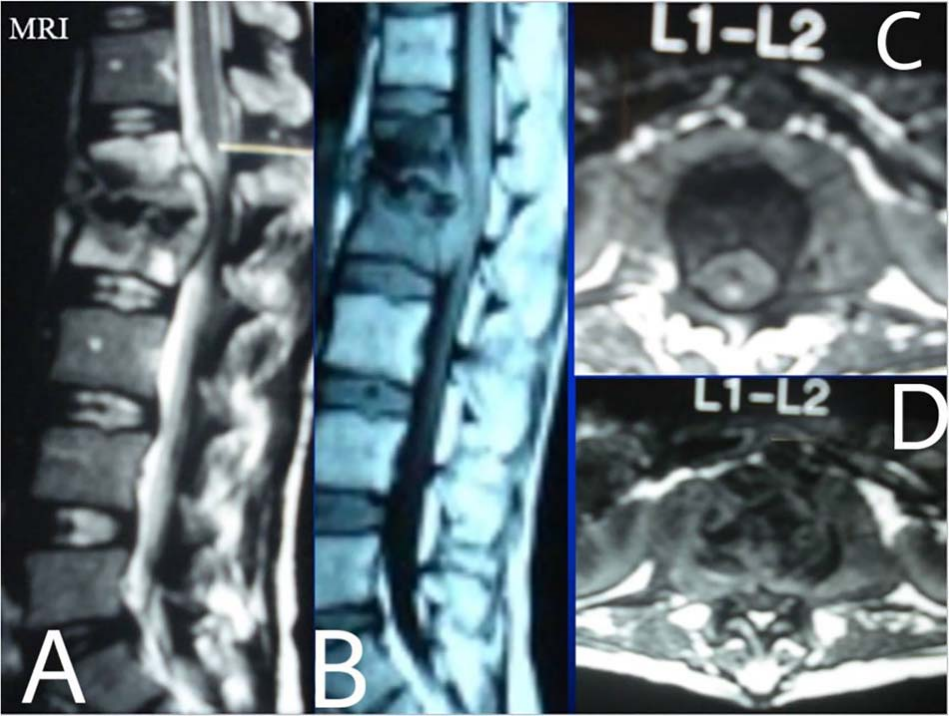
Lumbar T1 and T2 consistent with an abscess at the L1–2 vertebral level. (**A**) and (**B**) showed that the height of the L1 and L2 vertebral bodies was markedly reduced and the dural sac and spinal cord were severely compressed by an abscess posterior to the vertebral body. (**C** and **D**) Axial T1 shows a well-defined paraspinal abscess. The anterior epidural abscess compresses the spinal cord.

**Figure 4:**
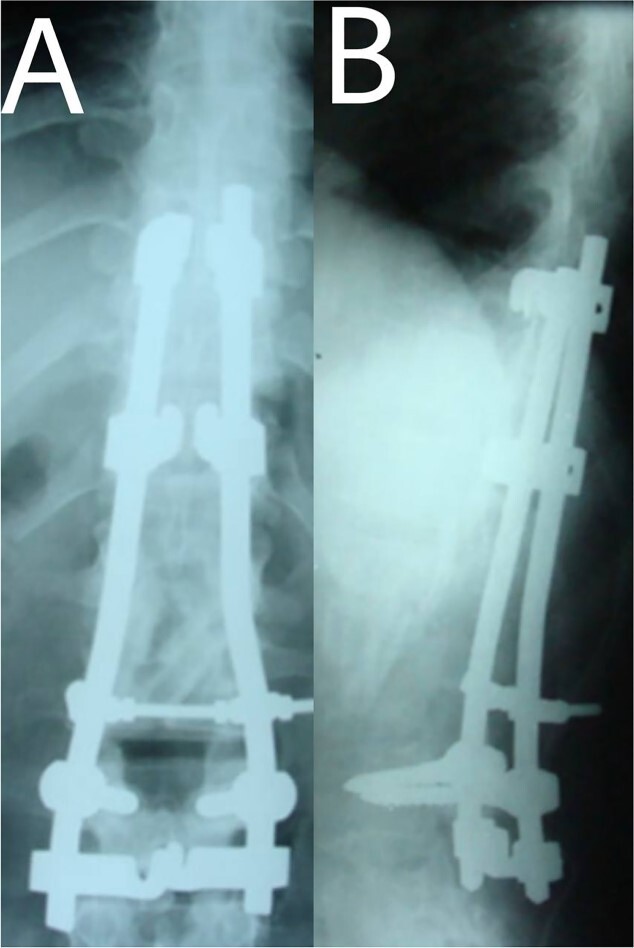
(**A**, **B**) Post-operation, anteroposterior and lateral view showing fibular autograft implantation followed by supplemental posterior instrumentation.

**Figure 5:**
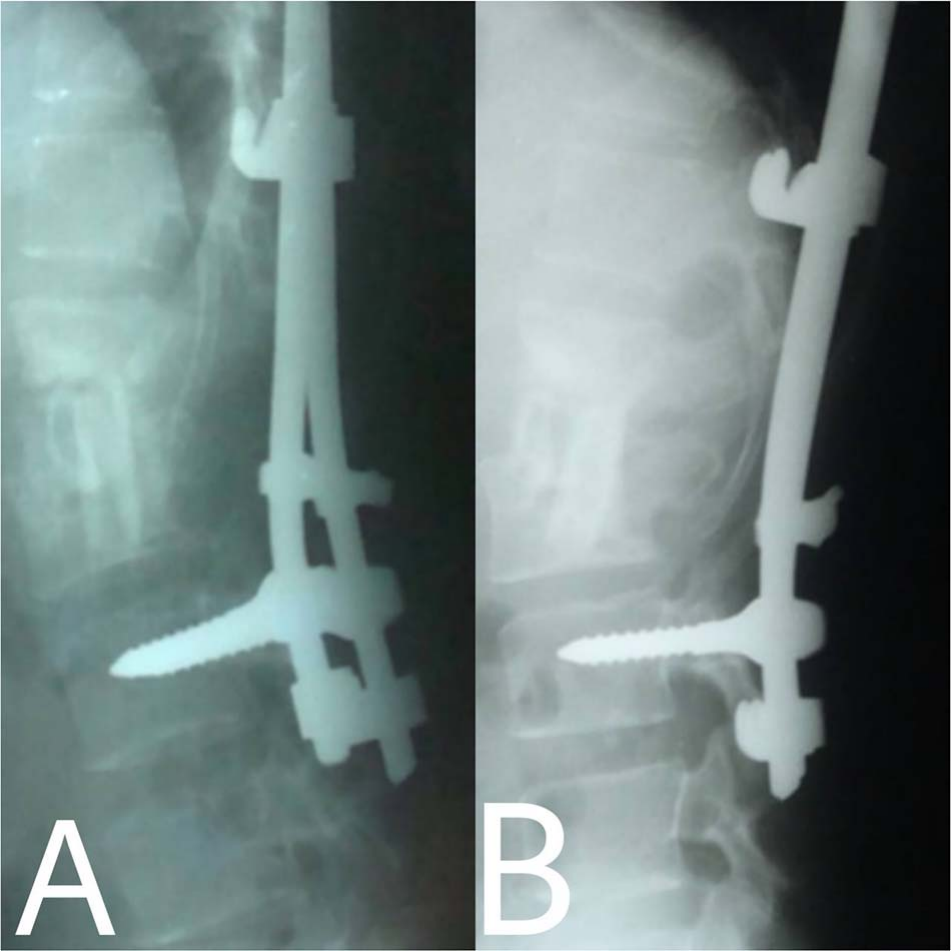
(**A**, **B**) 2, 4 months after single-stage posterior extensive debridement and fibular autograft implantation followed by supplemental posterior instrumentation performed to treat the spinal infection.

**Figure 6:**
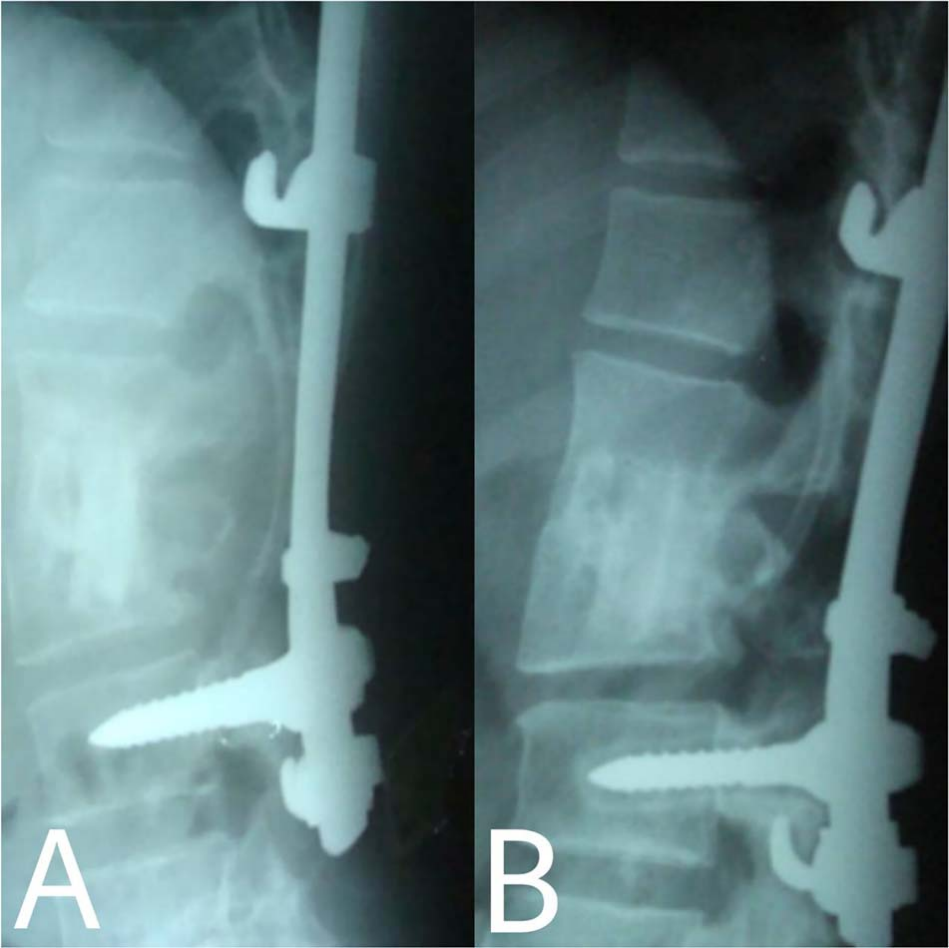
(**A**, **B**) Complete bony incorporation between the implanted fibular autograft and host vertebral body was noted on the lateral radiograph 1, and 14 years later.

A 24-year-old farmer female was admitted to the Department of Neurosurgery with low back pain, no fever, cough, motor deficit, or any symptoms. Medical, surgical, family histories and physical examinations were unremarkable. The laboratory findings showed a white cell count of 9.8 × 103/μL, C-reactive protein concentrations of 20, erythrocyte sedimentation rate of 50 mm/1 h, 90 mm/2 h and tuberculin skin test was positive. X-ray films showed the collapse of the L1, L2 vertebrae, and L1–2 intervertebral space (Fig. 1). An abscess was observed at the L1–L2 vertebral level in lumbar magnetic resonance imaging (MRI) (Fig. 2). Chest X-rays and Sputum smear were negative. The patient was treated with anti-TB treatment (Isoniazid, Rifampicin, Ethambutol, Pyrazinamide) due to spinal TB findings on MRI (Fig. 3). After 40 days, the patient was diagnosed with grade 1/5 lower limb weakness, and bladder and bowel dysfunction, with no impairment in sensation, which predicts spinal infection. MRI confirmed these abnormalities and showed typical findings such as vertebral endplate destruction, bone marrow and disk signal abnormalities, and paravertebral or epidural abscesses (Fig. 3). Due to clinical manifestations and MRI spinal TB findings (Fig. 3), the patient underwent surgical debridement, interbody fusion and internal fixation with fibular autografting and supplemental posterior spinal stabilisation using a posterior-only approach. On post-operative follow-up, the treatment continued for 9 months, in addition to physical therapy for lower limb weakness. In the end, the patient returned to full motion with grade 5/5 in the lower limb, normal sensation, and no bladder or bowel incontinence. No recurrence was observed in the grafting area. Radiologically 2, 6 and 12 months, 14 years’ post-operation, the patient had achieved full bony graft spinal fusion (Figs 4–6).

## DISCUSSION AND CONCLUSIONS

Even though a spinal epidural abscess is a rare infectious situation, its incidence is increasing due to risk factors such as chronic illnesses, immunodeficiency states and drug abuse. MRI is an effective diagnostic test for spinal infection and to differentiate between TB and pyogenic spondylitis. It is difficult to determine the diagnosis when there are atypical manifestations of infectious spondylitis. However, in our case, MRI demonstrated the typical finding such as the compression of the spinal cord by the two vertebral endplates destruction, the epidural abscess, the disk signal and bone marrow abnormalities. Conservative therapy is the basis of spinal TB treatment. This approach is insufficient in some cases that require surgical interventions [4]. When indicated, delayed surgical management may lead to a bad prognosis [5]. As in our case, the paraparesis developed after 40 days of TB drug administration. These aspects necessitated surgical procedures to prevent further spinal cord compression [6]. Surgical techniques included posterior approach decompression, abscess resection, bone grafting and instrumentations. Spinal TB primarily impacts the anterior elements due to its blood supply profusion. Hence, the conventional approach has long been to use the anterior approach in spinal TB treatment [6, 7]. A prospective study has shown that the anterior approach has good results in a minimum period of a 3-year follow-up. Also, Ge *et al* [8]. reported a case of spinal TB involving the L1 vertebra with a massive paravertebral abscess. They used the anterior approach in the decompression and grafting with posterior instrumentations and their results were satisfactory. However, in recent years, the posterior approach proved its advantages, including its familiarity, enough space for an adequate debridement of the focal lesion, in addition to less surgical invasion. Both approaches lead to reduced operation duration and fewer rates of complications [9]. On the other hand, the anterior approach has its risks, such as abdominal visceral injury, and neural and vascular injuries. Moreover, spinal instability may occur [10]. As for grafting, we used an autogenous bone graft from the fibula for osseous loss, which may be considered a golden standard, because of its immune correspondence and osteoinductive capability [4]. There are considerations about inserting a bone graft in an infected site, like the surrounding tissue’s capability to provide the biological background for recovery. The presence of biomechanical stability enables biological reactions to enhance bone ingrowth [11]. Bansal *et al.* reported the fibular strut graft efficacy along with cancellous graft in an anterior approach and without instrumentations [12]. Singh *et al.* also showed the same results but without cancellous graft [7]. In our case, the bone graft was supported with the posterior (T10–L3) hooks combination and screws. Spinal cord decompression is the priority. Besides, the infected extracted tissues had to be replaced with another component for the spinal column stability. The X-ray showed a successful fusion post-operation. The medication was administered for 9 months to prevent the recurrence of the infection [13]. After treatment, the muscle strength became 5/5 up from the pre-operative 1/5. Within 14 years of follow-up, the X-ray demonstrated a good bone fusion and the patient’s status was good (Fig. 6). Minimally invasive spinal surgery (MISS) has been used increasingly over the past two decades [14]. MISS is performed using smaller incisions instead of traditional open approaches to accomplish spine surgical operations. MISS includes spinal endoscopy and robotics, which has improved the accuracy of instrumentation placement and virtual/augmented reality that has been very helpful for practicing surgical skills without patients. These techniques decreased the sedation requirements, blood loss and hospitalisation time [15]. In conclusion, early diagnosis of TB spondylitis with proper treatment and surgical intervention immediately after a neurological deficit can protect the patients from the chronic spinal deformity. The posterior approach was sufficient and effective in our case. However, the surgical procedure should be planned on a case-by-case basis. Fibular graft and instrumentations were effective, although they were inserted on an infected background.

## Data Availability

All data generated during this study are included in this published article and its supplementary information files.
